# Dramatic response of a gastrointestinal stromal tumor to neadjuvant imatinib therapy

**DOI:** 10.1186/1477-7819-7-30

**Published:** 2009-03-16

**Authors:** Shohrat Annaberdyev, Joseph Gibbons, Jeffrey M Hardacre

**Affiliations:** 1 Case Western Reserve University School of Medicine, Cleveland, Ohio, USA; 2 Division of Hematology/Oncology, University Hospitals Case Medical Center, Cleveland, Ohio, USA; 3 Division of Surgical Oncology, University Hospitals Case Medical Center, Cleveland, Ohio, USA

## Abstract

Gastrointestinal stromal tumors (GISTs) are the most common sarcoma of the alimentary tract and are believed to derive from the interstitial Cell of Cajal. Imatinib mesylate (Gleevec^®^; Novartis, Basel, Switzerland) has revolutionized the treatment of GISTs and is generally used in the metastatic and adjuvant settings. We report the case of a 61-year old man who was treated with neoadjuvant imatinib for a massive gastric GIST with the hope of avoiding a potential multi-visceral resection.

## Case presentation

A 61-year old man presented with a left upper quadrant abdominal mass after experiencing several intermittent episodes of nausea, vague abdominal discomfort, and mild acid reflux. He also reported a nine kilogram weight loss over the prior six to eight months. Physical examination revealed a large mass in his upper abdomen.

**Figure 1:**
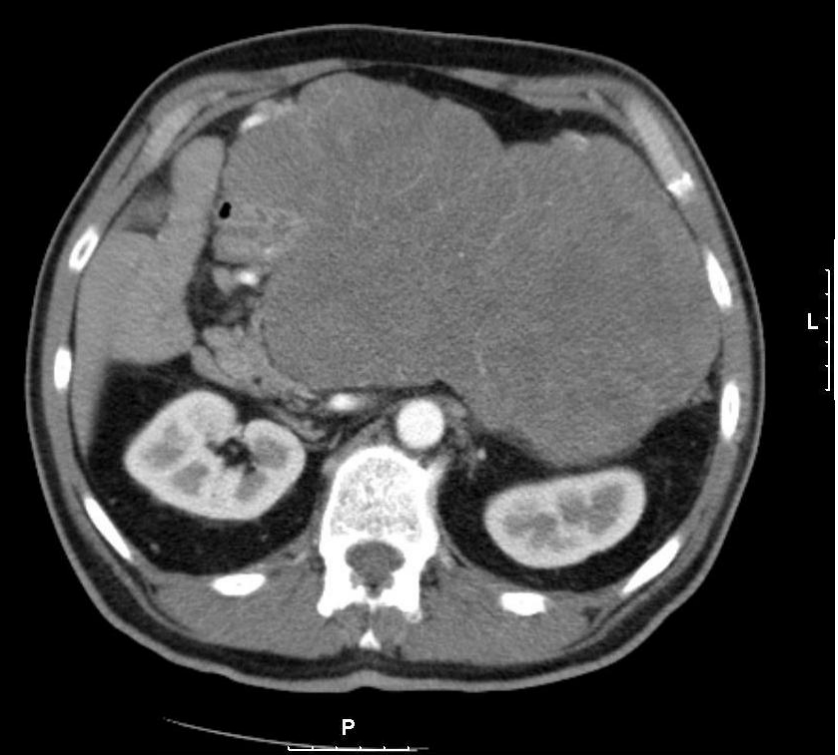
**Initial CT scan revealing an abdominal mass measuring 21 × 12 cm**.

Abdominal computed tomography (CT) revealed a 21 × 12 cm heterogeneous mass occupying his mid and left upper quadrants (Figure 1). Based on its location and imaging characteristics, the mass was hypothesized to be a GIST. The differential also included lymphoma, retroperitoneal sarcoma, and, less likely, a pancreatic neoplasm. To establish the diagnosis, an endoscopic ultrasound was performed and a core biopsy of the mass was obtained. The pathology of the core biopsy classified the mass as a spindle cell neoplasm that stained positive for CD117, consistent with a GIST.

Given the size and location of the lesion at the time of initial evaluation, resection of the mass would likely have necessitated a multi-visceral resection. Based on recent reports of effective preoperative imatinib therapy, a trial of neoadjuvant imatinib was felt to be the optimal treatment strategy to down-stage the tumor and minimize the extent of resection [1].

**Figure 2:**
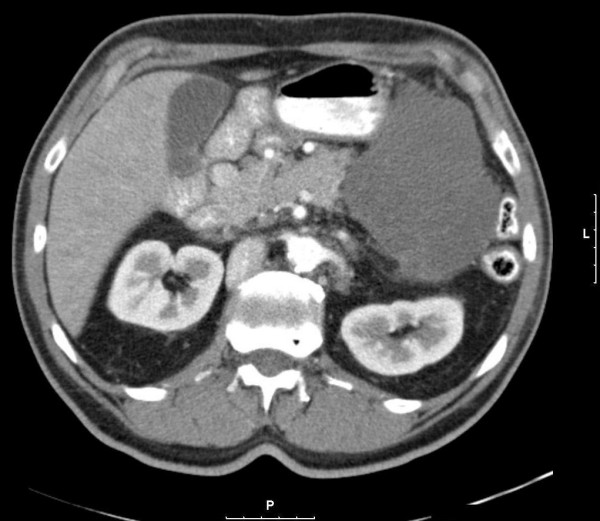
**Follow up CT scan after six months of Imatinib**. The tumor has shrunk to 10 × 8 cm.

The patient was treated with imatinib and tolerated the therapy well, with the exception of developing mild periorbital edema, the most commonly reported side effect of imatinib [2]. He was followed with CT scans performed at two-month intervals. The mass measured 21 × 12 cm on initial imaging. Subsequent measurements were 16.9 × 9.1 cm, 12.2 × 9.6 cm, and 10 × 8 cm (Figure 2) at two, four, and six month intervals, respectively. Upon reviewing the patient's imaging and clinical course after six months of treatment, it was felt that resection was appropriate. Further, there was concern regarding the development of secondary resistance to imatinib.

The patient was counseled regarding a likely partial gastrectomy but also informed that a total gastrectomy and even a multi-visceral resection may be needed. At operation, he was found to have a softball-sized mass attached by a stalk to his stomach. He underwent a wedge resection of his stomach that included the stalk and tumor en bloc. Pathological analysis revealed a tumor of 15 cm in greatest dimension. There were extensive areas of ischemic necrosis. There were up to two mitoses per 50 high-power fields. The margins of the gastric resection were free of neoplasm.

The patient recovered from the operation. At least one year of adjuvant imatinib therapy is planned.

## Discussion

GISTs are the most common sarcoma of the gastrointestinal tract. GISTs are characterized by the expression of the cell surface marker CD117, which is found in 95–100% of GISTs [3]. CD117 is a receptor tyrosine kinase, coded for by the c-kit proto-oncogene. Overexpression of this gene is most commonly caused by an activating mutation of exon 11 and is hypothesized to be responsible for GIST oncogenesis [4].

Historically, GISTs have responded very poorly to radiation and cytotoxic chemotherapy, making surgical resection the only effective treatment. Imatinib, a receptor tyrosine kinase inhibitor, was first used in the treatment of chronic myelogenous leukemia, which is characterized by the constitutive activation of the Bcr-Abl tyrosine kinase [5]. Imatinib's effect is mediated by its ability to bind to the ATP-binding site on the tyrosine kinase, thus preventing its function.

The first report of imatinib in the treatment of GIST was published in 2001 by Joensuu et al [6]. Since then, multiple studies have confirmed the usefulness of imatinib therapy in treating GISTs, leading to FDA approval of imatinib in the treatment of metastatic and/or unresectable GISTs [7]. Notably, Demetri et al conducted a study of 147 patients who received either 400 or 600 mg of imatinib daily. They reported a 54% radiographic response in their patient population [2].

The most common side effects of imatinib are fluid retention, diarrhea, nausea, fatigue, muscle cramps, abdominal pain, and rash. Extremity a facial edema (the latter was experienced by the patient in this case) were the most frequent adverse effects in the Demetri study.

A new approach is evolving that relies on neoadjuvant imatinib to down-stage the tumor in cases of advanced GISTs. Prior studies have described patients with inoperable or metastatic GISTs who underwent treatment with imatinib and had a dramatic response allowing surgical resection [8,9].

The median time for an objective response to imatinib is four months, but maximal response is reported to take six months or longer [10]. Response is defined as absence of progression at the time of first follow-up, generally two-three months after starting therapy. The patient in our case was believed to have achieved sufficient response to allow for an uncomplicated, successful resection of the GIST.

While most patients respond to imatinib, many eventually develop resistance. Initial (primary) resistance is defined as progression of disease at the time of first follow up after start of therapy [11]. These patients are not responsive to imatinib. Late (secondary) resistance is seen in a patient who experiences disease progression after a period of response. Patients who respond should be followed with serial imaging. Ideally, resection should be performed before the development of resistance.

The role of imatinib in the neoadjuvant setting is illustrated in this case. While the GIST found in this patient may not have been inoperable before imatinib treatment, the procedure may have required a multi-visceral resection. Neoadjuvant imatinib was given and after dramatic radiographic response, a simple wedge gastric resection was needed.

Surgeons should consider the use of neoadjuvant imatinib therapy in patients with marginally resectable GISTs. A response to imatinib can allow for a less extensive though still therapeutic oncologic resection.

## Competing interests

The authors declare that they have no competing interests.

## Authors' contributions

SA drafted the manuscript. JG reviewed an amended the manuscript. JMH reviewed and amended the manuscript. All authors read and approved the final manuscript.
